# Glucose impairs tamoxifen responsiveness modulating connective tissue growth factor in breast cancer cells

**DOI:** 10.18632/oncotarget.22552

**Published:** 2017-11-20

**Authors:** Maria Rosaria Ambrosio, Vittoria D’Esposito, Valerio Costa, Domenico Liguoro, Francesca Collina, Monica Cantile, Nella Prevete, Carmela Passaro, Giusy Mosca, Michelino De Laurentiis, Maurizio Di Bonito, Gerardo Botti, Renato Franco, Francesco Beguinot, Alfredo Ciccodicola, Pietro Formisano

**Affiliations:** ^1^ Department of Translational Medicine, University of Naples “Federico II” & URT “Genomic of Diabetes” of Institute of Experimental Endocrinology and Oncology “G. Salvatore”, National Council of Research (CNR), Naples 80131, Italy; ^2^ Institute of Genetic and Biophysics “A. Buzzati-Traverso”, CNR, Naples 80131, Italy; ^3^ Pathology Unit, National Cancer Institute “G. Pascale Foundation”, Naples 80131, Italy; ^4^ Department of Breast Surgery and Cancer Prevention, National Cancer Institute “G. Pascale Foundation”, Naples 80131, Italy; ^5^ Pathology Unit, Università degli Studi della Campania “Luigi Vanvitelli”, Naples 80138, Italy; ^6^ Department of Science and Technology, University of Naples “Parthenope”, Naples 80131, Italy

**Keywords:** breast cancer, tamoxifen, glucose, adipose tissue, connective tissue growth factor

## Abstract

Type 2 diabetes and obesity are negative prognostic factors in patients with breast cancer (BC). We found that sensitivity to tamoxifen was reduced by 2-fold by 25 mM glucose (High Glucose; HG) compared to 5.5 mM glucose (Low Glucose; LG) in MCF7 BC cells. Shifting from HG to LG ameliorated MCF7 cell responsiveness to tamoxifen. RNA-Sequencing of MCF7 BC cells revealed that cell cycle-related genes were mainly affected by glucose. Connective Tissue Growth Factor (CTGF) was identified as a glucose-induced modulator of cell sensitivity to tamoxifen. Co-culturing MCF7 cells with human adipocytes exposed to HG, enhanced CTGF mRNA levels and reduced tamoxifen responsiveness of BC cells. Inhibition of adipocyte-released IL8 reverted these effects. Interestingly, CTGF immuno-detection in bioptic specimens from women with estrogen receptor positive (ER^+^) BC correlated with hormone therapy resistance, distant metastases, reduced overall and disease-free survival. Thus, glucose affects tamoxifen responsiveness directly modulating CTGF in BC cells, and indirectly promoting IL8 release by adipocytes.

## INTRODUCTION

Breast cancer (BC) is the most common female malignant neoplasia with the highest incidence in the industrialized world [1]. Type 2 diabetes (T2D) is associated with a 20% increased risk of BC and a more aggressive phenotype [2, 3]. Moreover, up to 16% of BC patients suffer from T2D or impaired glucose tolerance [4]. Notably, hyperglycemia is correlated with a poorer therapeutic outcome and reduced drug response in BC [5, 6, 7].

Enhanced glucose uptake is a well-known metabolic hallmark of cancer cells [8]. Indeed, the anabolic urge of tumor cells drives them to adapt with profound metabolic changes, among which the most remarkable is known as the ‘Warburg effect’ [9]. Thus, hyperglycemia may be responsible for the excess of glucose supply for glucose-hungry cancer cells, also contributing to a more aggressive phenotype [10]. A possible effect of diabetes-related hyperglycemia on the outcome of chemotherapy for BC should not be neglected.

Besides acting on cancer cells, glucose may affect surrounding stromal cells, which in turn may interfere with anti-cancer drug response. The interactions between tumor cells and the associated stroma represent a powerful relationship that influences disease initiation, progression and patient prognosis [11, 12]. Consistently, the number of agents entering clinical trials that specifically target interactive pathways between neoplastic and stromal cells has increased [13]. A highly relevant component of tumor microenvironment in mammary gland is represented by adipose tissue [14]. Given the abundance and the proximity of adipocytes with tumor cells, they have been demonstrated to integrate inputs from the metabolic environment and promote growth and invasiveness of BC cells [15, 16]. Notably, recent studies suggested the active role of adipocytes in BC resistance to chemotherapy [17].

During the last decade, the significant reduction of the mortality for hormone-dependent BC is thought to be attributable to the widespread use of tamoxifen, a selective estrogen receptor modulator [18, 19, 20]. However, chemotherapy resistance continues to be a major problem in the treatment of cancer patients. Several studies have already revealed a number of mechanisms underlying tamoxifen resistance [21, 22, 23]. Nevertheless, understanding the molecular mechanisms and pathways involved in tamoxifen resistance is of huge clinical importance and could result in novel strategies to improve survival of diabetic patients with breast cancer.

## RESULTS

### Glucose impairs MCF7 cell responsiveness to tamoxifen

**Figure 1:**
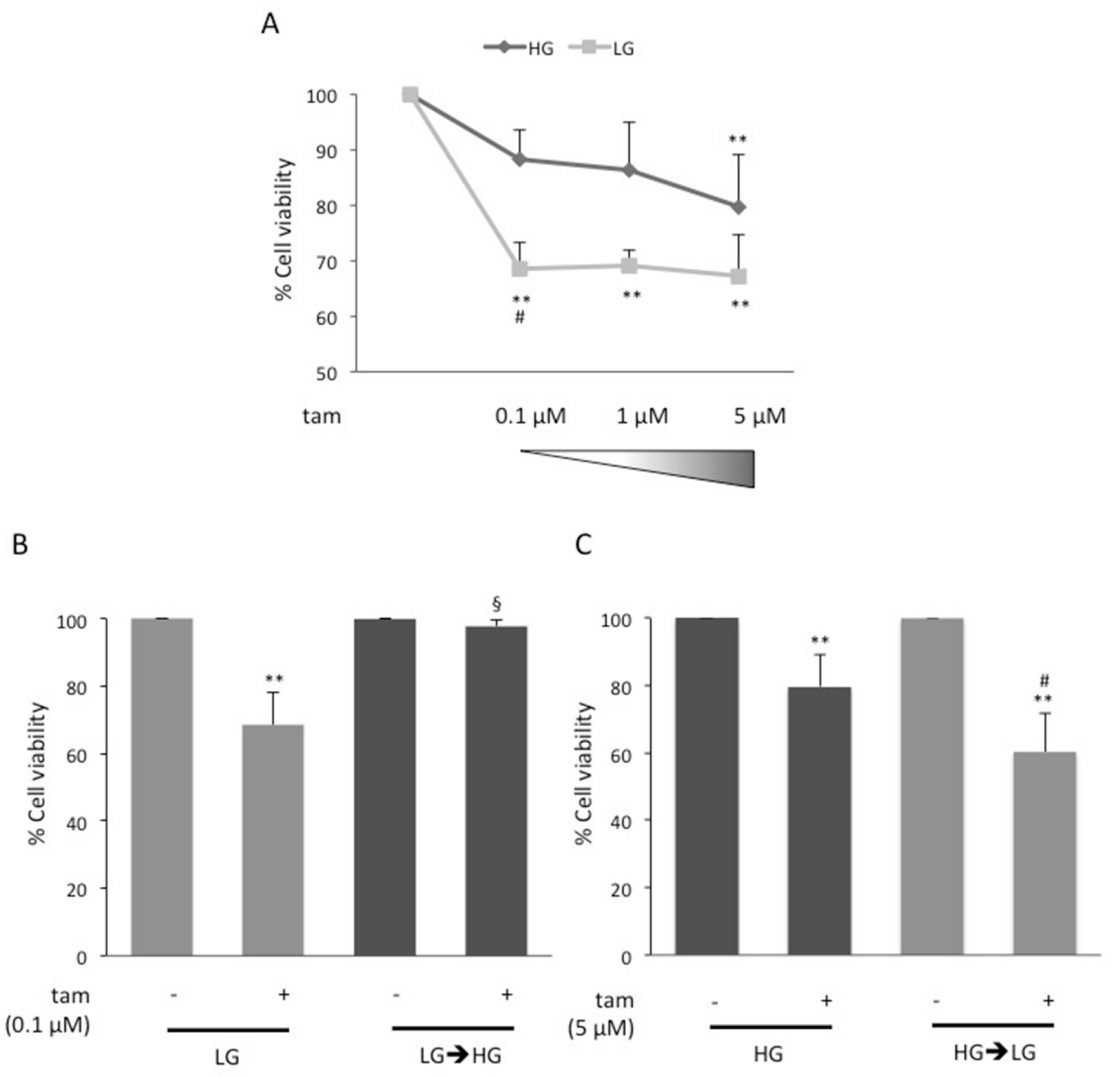
Effect of glucose on MCF7 cell responsiveness to tamoxifen **(A)** MCF7 cells grown in high glucose (25mM; HG) or in low glucose (5.5mM; LG), were treated with estradiol (100nM; E_2_) and raising concentration (0.1μM, 1μM, 5μM) of tamoxifen (tam); **(B)** LG cells were shifted to high glucose (LG→HG) during the treatment with E_2_ and 0.1μM tam; **(C)** HG cells were shifted in low glucose (HG→LG) when treated with E_2_and 5μM tam. For all the panels (A), (B) and (C), cell viability was assessed, after four days, by sulforhodamine B assay (see Methods). The results were reported as percentage of viable cells compared to positive control (cells treated with E_2_ alone), considered as maximum viability (100%). Data represent the mean ± SD of at least three independent triplicate experiments. ^*^ denote statistically significant values compared with positive control (^**^p<0.01); ^§^ denote statistically significant values compared with tam treatment in LG cells (^§^p<0.01). ^#^ denote statistically significant values compared with tam treatment in HG cells (^#^p<0.05). See also Supplementary Figure 1.

The effect of glucose on BC cell response to tamoxifen was addressed. MCF7 cells were cultured in 5.5mM glucose (Low Glucose; LG), corresponding to normal fasting glucose levels in humans, or in 25mM glucose (High Glucose; HG) corresponding to the regular culture condition for this cell line but resembling hyperglycemia in humans. As expected, cell proliferation rate was increased in HG compared to LG condition (data not shown). Thus, all further cell viability assays described were normalized *per* condition (HG or LG; see figure legends). The cells were treated with raising concentrations (0.1μM, 1μM and 5μM) of tamoxifen. As reported in Figure 1A, upon the treatment with the lower tamoxifen doses (0.1μM, 1μM), cell viability was significantly reduced in LG (≈30%; p<0.01), and not in HG, compared to positive control (Figure 1A). Interestingly, shifting LG cells to HG (LG→HG) during tamoxifen treatment (0.1μM) leads to a significant reduction of drug effect on cell viability (Figure 1B). Conversely, only the highest tamoxifen dose (5μM) significantly reduced cell viability in HG (≈20%; p<0.01; Figure 1A). Of note, shifting HG cells to LG (HG→LG), ameliorated tamoxifen responsiveness determining a significant reduction of cell viability (≈40%; p<0.01; Figure 1C). No difference in the levels of estrogen receptor (ER) was observed in both conditions (Supplementary Figure 1A).

### RNA-Seq identifies CTGF as a glucose-induced factor that impairs MCF7 cell sensitivity to tamoxifen

**Figure 2:**
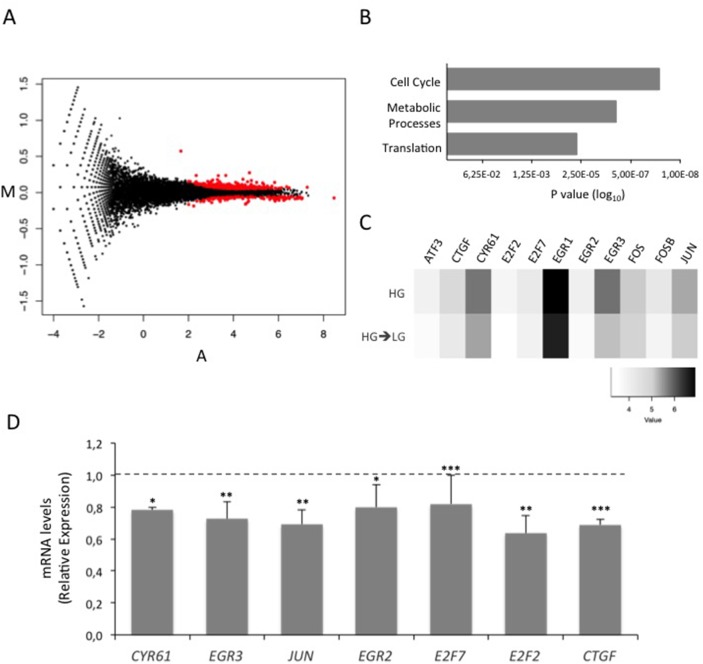
Effect of glucose on MCF7 cell transcriptome **(A)** Standard MA plot (M, log ratios and A, mean average) of normalized (upper quartile) RNA-Seq data for each RefSeq gene. The y-axis reports logFC (fold-change), the x-axis the average log intensity (A) for each gene (indicated as dots). Red dots indicate genes that differentially expressed (P≥0.8) in the HG vs HG->LG comparison. **(B)** Bar graph showing the most significant gene pathways enriched with DEGs after the shift of MCF7 cells from high glucose (25mM; HG) to low glucose (5.5mM; HG→LG). **(C)** Heatmap of log-transformed normalized gene expression values for a small subset of highly significant deregulated genes belonging to “Cell cycle” pathway. **(D)** MCF7 cells grown HG (dotted line) were shifted in LG (HG→LG) during the treatment with estradiol (100nM; E_2_). After four days, mRNA expression levels of “Cell cycle” DEGs were determined by qRealTime-PCR (see Methods and Table 1). Data were normalized on Hypoxanthine Guanine Phosphoribosyl Transferase (*HPRT*) gene as internal standard. Bars represent the mean ± SD of three independent triplicate experiments and show the mRNA levels of DEGs in HG→LG cells relative to those in HG cells. ^*^ denote statistically significant values (^*^p<0.05, ^**^p<0.01, ^***^p<0.001). See also Supplementary Figure 1.

**Figure 3:**
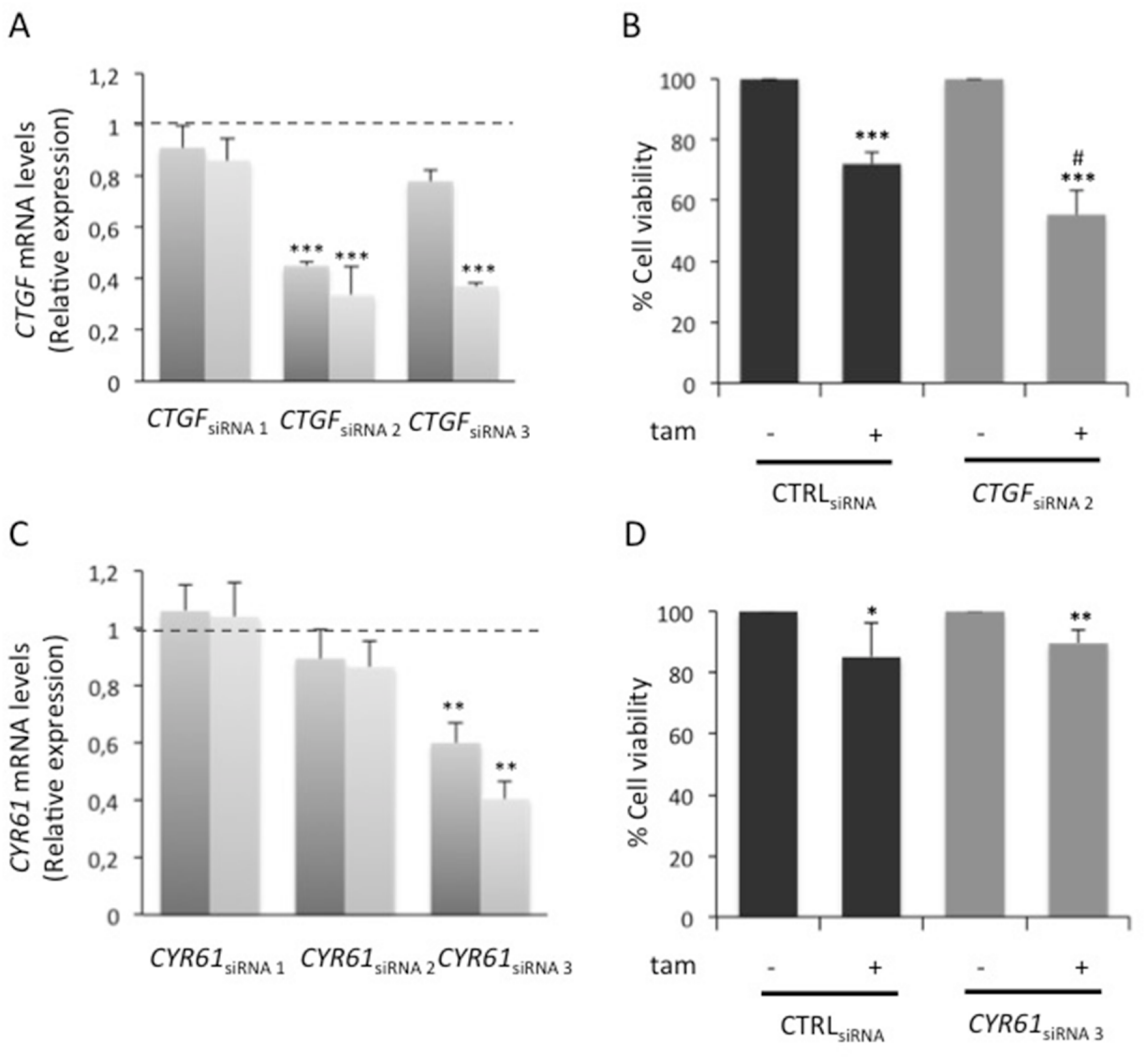
Role of glucose-induced CTGF on HG cell responsiveness to tamoxifen **(A)** MCF7 cells cultured in high glucose medium (25mM; HG) were transfected (see Methods) with three different siRNAs recognizing *CTGF* (10nM; *CTGF*_siRNA_) or with a control siRNA (10nM; CTRL_siRNA_); **(C)** HG cells were transfected with three different siRNAs recognizing *CYR61* (1nM; *CYR61*_siRNA_ cells) or with a control siRNA (1nM; CTRL_siRNA_). For both panels (A) and (C), after 6 hours, the cells were feed with complete medium and incubated for further 24 or 48 hours. The efficiency of siRNAs activity was assessed by qRealTime-PCR, as described in Methods. Data were normalized on Hypoxanthine Guanine Phosphoribosyl Transferase (*HPRT)* gene as internal standard. Bars represent the mean ± SD of three independent triplicate experiments and show the mRNA expression levels of (A) *CTGF* in *CTGF*_siRNA_ cells and (C) *CYR61* in *CYR61*_siRNA_ cells, both relative to those in CTRL_siRNA_ cells (dotted line), after 24 (dark grey columns) and 48 hours (light grey columns) transfection. ^*^ denote statistically significant values (^**^p<0.01; ^***^p<0.001). **(B)** HG cells transfected with selected CTGF siRNA (*CTGF*_siRNA 2_) or with control siRNA (CTRL_siRNA_) were treated with E_2_ (100nM) and tam (5μM); **(D)** HG cells transfected with selected CYR61 siRNA (*CYR61*_siRNA 3_) and with control siRNA (CTRL_siRNA_) were treated with E_2_ and tam (5μM). For both panels (B) and (D), as positive control, the cells were treated with E_2_ alone. After 24 hours, cell viability was determined, by sulforhodamine B assay (see Methods). The results were reported as percentage of viable cells compared with positive control considered as 100% viability. Data represent the mean ± SD of three independent triplicate experiments. ^*^ denote statistically significant values compared with positive control (^*^p<0.05, ^**^p<0.01, ^***^p<0.001); # denote statistically significant values compared with tam treatment in CTRL_siRNA_ cells (^#^p<0.01).

**Figure 4:**
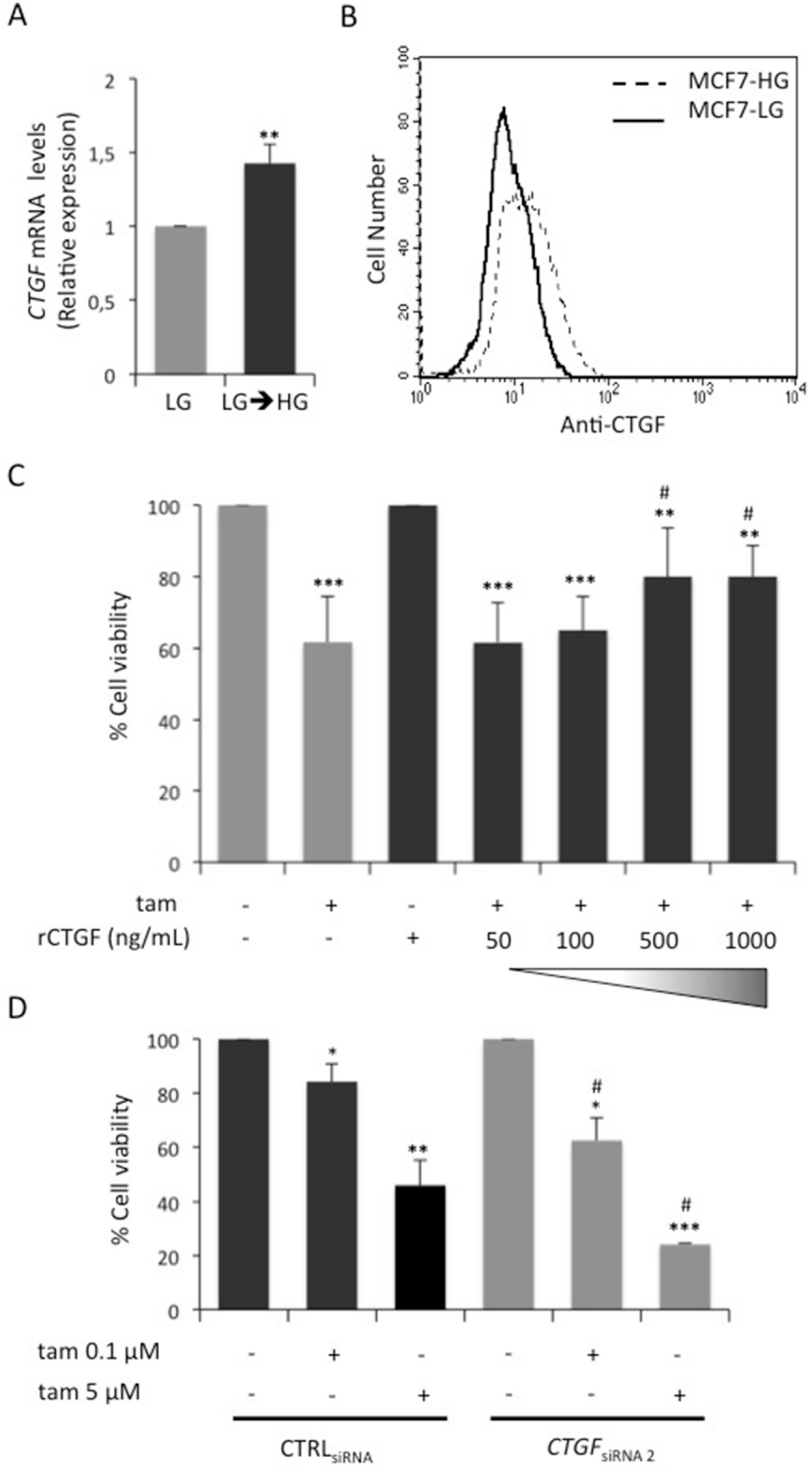
Role of glucose-induced CTGF on LG cell responsiveness to tamoxifen **(A)** MCF7 cells grown in low glucose medium (5.5mM; LG) were shifted in high glucose medium (LG→HG) during the treatment with E_2_ (100nM). After four days, mRNA expression levels of *CTGF* were determined by qRealTime-PCR (see Methods). Data were normalized on *HPRT* gene as internal standard. Bars represent the mean ± SD of three independent triplicate experiments and show the mRNA levels of *CTGF* in LG→HG cells relative to those in LG cells. ^*^ denote statistically significant values (^**^p<0.01). **(B)** CTGF protein levels in MCF7 chronically exposed to high (HG 25mM - dotted line) or low (LG 5.5mM - solid line) glucose concentrations. One representative experiment is shown. **(C)** LG cells were treated with E_2_ and tam (5μM) in presence or absence of human recombinant CTGF protein at increasing doses (50 ng/mL, 100 ng/mL, 500 ng/mL, 1000 ng/mL; rCTGF). As positive control, the cells were treated with E_2_ alone. After 4 days, cell viability was assessed by sulforhodamine B assay, as described in Methods. The results were reported as percentage of viable cells compared with positive control, considered as 100% viable cells. Data represent the mean ± SD of three independent triplicate experiments. ^*^ denote statistically significant values compared with positive control (^**^p<0.01;^***^p<0.001); ^#^ denote statistically significant values compared with tam treatment in untreated cells (^#^p<0.05). See also Supplementary Figure 1. **(D)** LG cells transfected with selected CTGF siRNA (*CTGF*_siRNA 2_) or with control siRNA (CTRL_siRNA_) were treated with E_2_ (100nM) and tam (0.1μM and 5μM). As positive control, the cells were treated with E_2_ alone. After 24 hours, cell viability was determined, by sulforhodamine B assay (see Methods). The results were reported as percentage of viable cells compared with positive control considered as 100% viability. Data represent the mean ± SD of three independent triplicate experiments. ^*^ denote statistically significant values compared with positive control (^*^p<0.05, ^**^p<0.01, ^***^p<0.001); ^#^ denote statistically significant values compared with tam treatment in CTRL_siRNA_ cells (^#^p<0.01).

RNA-Seq was used to evaluate global changes in the transcriptome of HG and HG→LG BC cells (GEO accession no. GSE97647). Interestingly, a variation in the expression levels of about 500 genes (Figure 2A and Supplementary Figure 1B) was observed upon glucose lowering. In detail, 310 and 184 genes were up- and down-regulated, respectively when the cells were shifted to LG. Enrichment analysis revealed that 70 differentially expressed genes (DEGs) belong to “Cell cycle” pathway (Figure 2B). Eleven out of 70 cell cycle-related DEGs - that displayed a more robust alteration after cell shift (Posterior probability ≥ 0.8) - were selected for further validation (Figure 2C). Remarkably, the significant down-regulation observed by RNA-Seq was confirmed for 7 out of 11 genes in three independent experiments (Figure 2D). RNA-Seq data and independent confirmatory experiments indicated that *CTGF* and *CYR61* - immediate-early genes of the CCN family - were significantly down-modulated upon the exposure to LG. Their possible contribution to MCF7 cell sensitivity to tamoxifen was hypothesized because they encode growth factors - that mediate early response to external *stimuli* - whose expression levels have been associated with breast cancer progression [24]. Interestingly, *CTGF* knockdown in HG cells (*CTGF*_siRNA_ cells; Figure 3A and Supplementary Figure 1C) significantly increased BC cell sensitivity to tamoxifen (≈40% cell viability; p<0.001; Figure 3B). A similar effect on cell viability was not determined by tamoxifen upon *CYR61* knockdown (*CYR61*_siRNA_ cells; Figure 3C-3D). Moreover, *CTGF* knockdown in HG cells did not affect the expression of the other “Cell cycle”-related DEGs, indicating that they may act independently (Supplementary Figure 1D). Overall, the increase in drug responsiveness observed in *CTGF*_siRNA_ cells was comparable to the effect of glucose lowering (see Figure 1C). Accordingly, the shift of tamoxifen-sensitive LG cells to HG induced *CTGF* mRNA expression (p<0.01; Figure 4A). Higher levels of CTGF protein in HG cells (compared to LG cells) were also observed (Figure 4B). Such glucose-mediated induction of *CTGF* further confirmed the RNA-Seq data (Supplementary Figure 1E). To assess if CTGF controls the sensitivity of LG cells to tamoxifen, MCF7 cells were treated with the drug in presence (or absence) of raising concentration of human recombinant CTGF protein (rCTGF). 500 ng/mL rCTGF significantly reduced tamoxifen responsiveness of LG cells (Figure 4C) to levels similar to those detected when LG cells were shifted to HG (see Figure 1B). At variance, lower rCTGF doses (50 and 100 ng/mL) had no effect (Figure 4C). Accordingly, *CTGF* knockdown in LG cells (*CTGF*_siRNA_ cells; Figure 4D) significantly increased BC cell sensitivity to tamoxifen (0.1μM and 5μM, ≈40% and ≈75% cell viability, respectively; Figure 4D).

### CTGF mediates glucose-induced adipocyte effect on MCF7 cell responsiveness to tamoxifen

**Figure 5:**
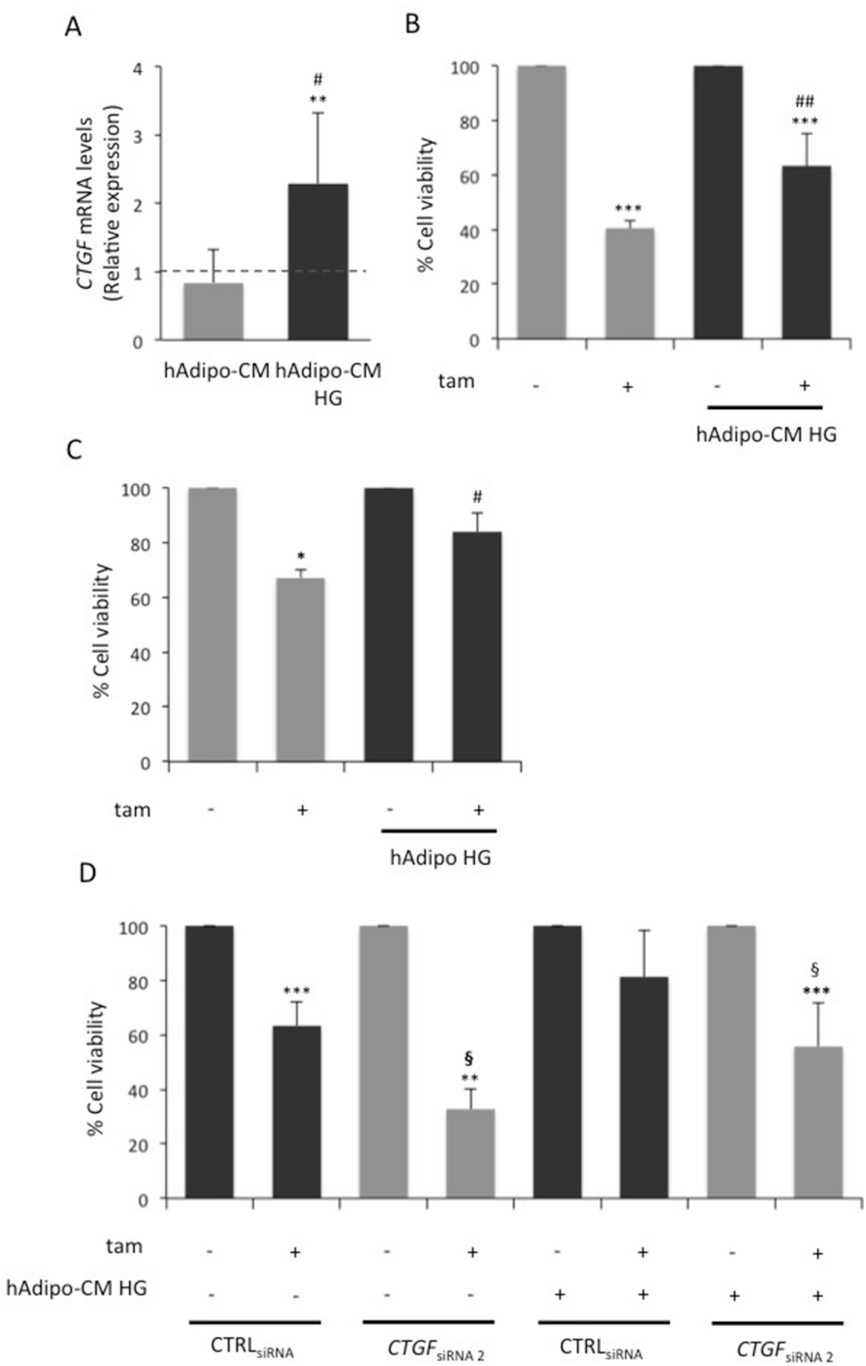
Role of CTGF in glucose-induced adipocyte-effect on MCF7 responsiveness to tamoxifen **(A)** Mature adipocytes, cultured in 15mM glucose, were incubated in serum free high glucose medium (25 mM; HG). After 8 hours, media were collected - from adipocytes pre-incubated or not in HG (hAdipo-CM and HG hAdipo-CM, respectively) - and applied onto MCF7 cells. As control, the cells were incubated in serum-free medium (15mM and 25mM, respectively). After four days, *CTGF* mRNA expression levels were determined by qRealTime-PCR, as described in Methods. Data were normalized on Hypoxanthine Guanine Phosphoribosyl Transferase (*HPRT)* gene as internal standard. Bars represent the mean ± SD of three independent triplicate experiments and show the expression levels of *CTGF* in cells treated with conditioned media relative to those in untreated cells (dotted line). ^*^ denote statistically significant values compared with positive control (^**^p<0.01); ^#^ denote statistically significant values compared with cells treated with hAdipo-CM (^#^p<0.05). **(B)** MCF7 cells were treated with estradiol (100nM; E_2_) and tamoxifen (5μM; tam) in presence of HG hAdipo-CM or in absence of it (in serum-free HG medium). As positive control, the cells were treated with E_2_ alone. After 4 days, cell viability was determined by sulforhodamine B assay, as described in Methods. **(C)** MCF7 cells were seeded in the upper chamber of a transwell system with or without mature adipocytes in the lower chamber. MCF7 cells were treated with E_2_ and tam (5μM) in serum free HG medium. As positive control, the cells were treated with E_2_ alone. After 24 hours, cell viability was assessed by crystal violet, as described in Methods. **(D)** MCF7 cells transfected (see Methods) with selected siRNAs recognizing *CTGF* (10nM; *CTGF*_siRNA 2_) or with a control siRNA (10nM; CTRL_siRNA_) were treated with E_2_ (100nM) and tam (5μM) in presence or absence of HG hAdipo-CM. As positive control, the cells were treated with E_2_ alone. After 24 hours, cell viability was assessed by sulforhodamine B assay, as described in Methods. For the panels (B), (C) and (D), the results were reported as percentage of viable cells compared with positive control, considered as 100% viable cells. Data represent the mean ± SD of three independent triplicate experiments. ^*^ denote statistically significant values compared with positive control (^*^p<0.05; ^***^p<0.001); ^#^ denote statistically significant values compared with tam treatment in absence of (B) hAdipo-CM, (C) adipocytes (^#^p<0.05; ^##^p<0.01); § denote statistically significant values compared with tam treatment in CTRL_siRNA_ cells (^§^p<0.05).

It is known that in BC the epithelial and stromal compartments communicate each other through soluble factors. In particular, since BC cells are embedded in adipocyte-rich microenvironment and glucose is able to modify the secretory capability of adipocytes [15, 16], the impact of glucose-induced adipocyte factors on both *CTGF* levels and tamoxifen responsiveness in BC cells was addressed. As expected [16], cell proliferation rate was increased in presence of conditioned media (CM) collected from adipocytes (data not shown). Thus, all further cell viability assays described were normalized *per* condition (presence and absence of CM; see figure legend). Interestingly, CM collected from adipocytes pre-incubated in HG (HG hAdipo-CM) increased by 2-fold *CTGF* expression levels in BC cells either compared to un-conditioned HG medium (p<0.01; Figure 5A) or compared to CM collected from adipocytes incubated in regular medium (hAdipo-CM; p<0.05; Figure 5A). Moreover, cell sensitivity to tamoxifen was worsened in presence of HG hAdipo-CM (p<0.01; Figure 5B) and almost completely abolished when BC cells were co-cultured with mature adipocytes in HG (HG hAdipo) (Figure 5C). No effects on *CTGF* expression and tamoxifen responsiveness were observed in MCF7 cells by using CM collected from adipocytes pre-incubated in LG (data not shown). *CTGF* knockdown in HG cells (*CTGF*_siRNA_ cells) significantly increased their sensitivity to tamoxifen (Figure 3B and 5D). Of note, HG hAdipo-CM did not retain their ability to reduce tamoxifen responsiveness upon *CTGF* knockdown in MCF7 cells, confirming that glucose-induced adipocyte-released molecules effect on tamoxifen sensitivity occurs through *CTGF* (Figure 5D).

### Adipocyte-released IL8 impairs MCF7 cell responsiveness to tamoxifen via modulation of CTGF

**Figure 6:**
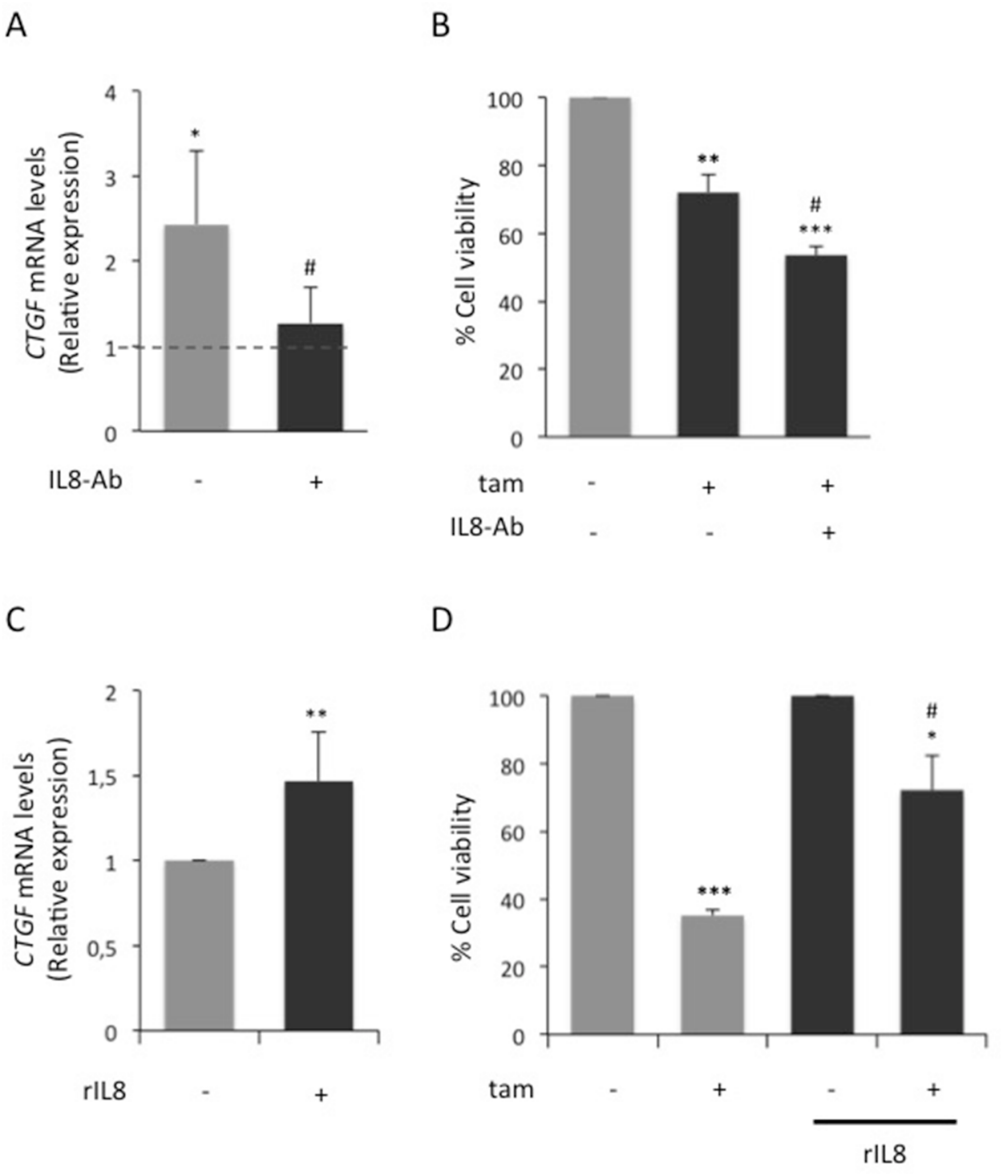
Effect of adipocyte-released IL8 on MCF7 cell breast tamoxifen responsiveness **(A)** Mature adipocytes were incubated in serum free high glucose medium (25mM; HG). After 8 hours, media were collected (HG hAdipo-CM) and applied onto MCF7 cells in presence or absence of IL8 blocking antibody (1μg/ml; IL8-Ab). As control, the cells were incubated in serum-free HG medium. After four days, *CTGF* mRNA expression levels in MCF7 cells were determined by qRealTime-PCR, as described in Methods. Data were normalized on Hypoxanthine Guanine Phosphoribosyl Transferase (*HPRT)* gene as internal standard. Bars represent the mean ± SD of three independent triplicate experiments and show the expression levels of CTGF compared to those in control cells (dotted line). ^*^ denote statistically significant value compared with control (^*^p<0.05); ^#^ denote statistically significant values compared with HG hAdipo-CM treatment in absence of IL8-Ab (^#^p<0.05). **(B)** MCF7 cells were treated with estradiol (100nM, E_2_) and tamoxifen (5μM; tam) in presence of HG hAdipo-CM, with or without IL8-Ab (1μg/ml; IL8-Ab). As positive control, the cells were treated with E_2_ alone. After 4 days, cell viability was determined by sulforhodamine B assay, as described in Methods. The results were reported as percentage of viable cells compared with positive control, considered as 100% viable cells. Data represent the mean ± SD of three independent triplicate experiments. ^*^ denote statistically significant values compared with positive control (^**^p<0.01; ^***^p<0.001);^#^ denote statistically significant values compared with tam treatment in presence of HG hAdipo-CM (^#^p<0.05). **(C)** MCF7 cells were incubated in presence or absence of human recombinant IL8 protein (1μg/ml; rIL8). After four days, *CTGF* mRNA expression levels were determined by qRealTime-PCR, as described in Methods. Data were normalized on *HPRT* gene as internal standard. Bars represent the mean ± SD of three independent triplicate experiments and show the expression levels of *CTGF* in cells treated with rIL8 relative to those in untreated cells. ^*^ denote statistically significant values (^**^p<0.01). **(D)** MCF7 cells were treated with E_2_and tam (5μM) in presence or absence of rIL8 (1μg/ml). As positive control, the cells were treated with E_2_ alone. After 4 days, cell viability was determined by sulforhodamine B assay (see Methods). The results were reported as percentage of viable cells compared with positive control, considered as 100% viable cells. Data represent the mean ± SD of three independent triplicate experiments. ^*^ denote statistically significant values compared with positive control (^*^p<0.05; ^***^p<0.001); ^#^ denote statistically significant values compared tam treatment in absence of rIL8 (^#^p<0.05). See also Supplementary Figures 2 and 3.

In adipocytes, glucose increases the release of IL8, CCL5 and IGF1 [15]. The effect of such factors on both *CTGF* expression and tamoxifen responsiveness was investigated. In presence of HG hAdipo-CM, the inhibition of IL8 by a blocking Ab prevented - at least in part - the induction of *CTGF* (Figure 6A), whereas CCL5 or IGF1 inhibition did not (Supplementary Figure 2A). Moreover, no significant changes were observed in tamoxifen responsiveness when adipocyte-released CCL5 or IGF1 were inhibited (Supplementary Figure 2B and 2C). IL8 inhibition was able to partially restore cell responsiveness to tamoxifen, significantly reducing the hAdipo-CM-induced effect on cell viability (p<0.05; Figure 6B). Consistently, the treatment of MCF7 cells with human recombinant IL8 protein (rIL8) induced *CTGF* mRNA expression (p<0.01; Figure 6C) and significantly reduced cell sensitivity to tamoxifen (p<0.05; Figure 6D) mimicking the hAdipo-CM-mediated effect. Notably, variation of glucose levels did not affect *CTGF* expression in adipocytes (Supplementary Figure 3A). On the other hand, neither the reduction or the increase of glucose concentration affected *IL8* expression in MCF7 (Supplementary Figure 3B). Also, *CTGF* knockdown did not affect IL8 mRNA expression in MCF7 (Supplementary Figure 3C). Accordingly, the addition of human rCTGF to MCF7 did not induce IL8 expression (Supplementary Figure 3C). Finally, whether CTGF may regulate IL8 expression in adipocytes was assessed. Interestingly, the treatment of mature adipocytes with human rCTGF induced a significant increase of *IL8* expression (Supplementary Figure 3D), suggesting a cell-specific response to CTGF signaling.

### CTGF levels in (ER^+^) BC tissues correlate with hormone therapy resistance

**Figure 7:**
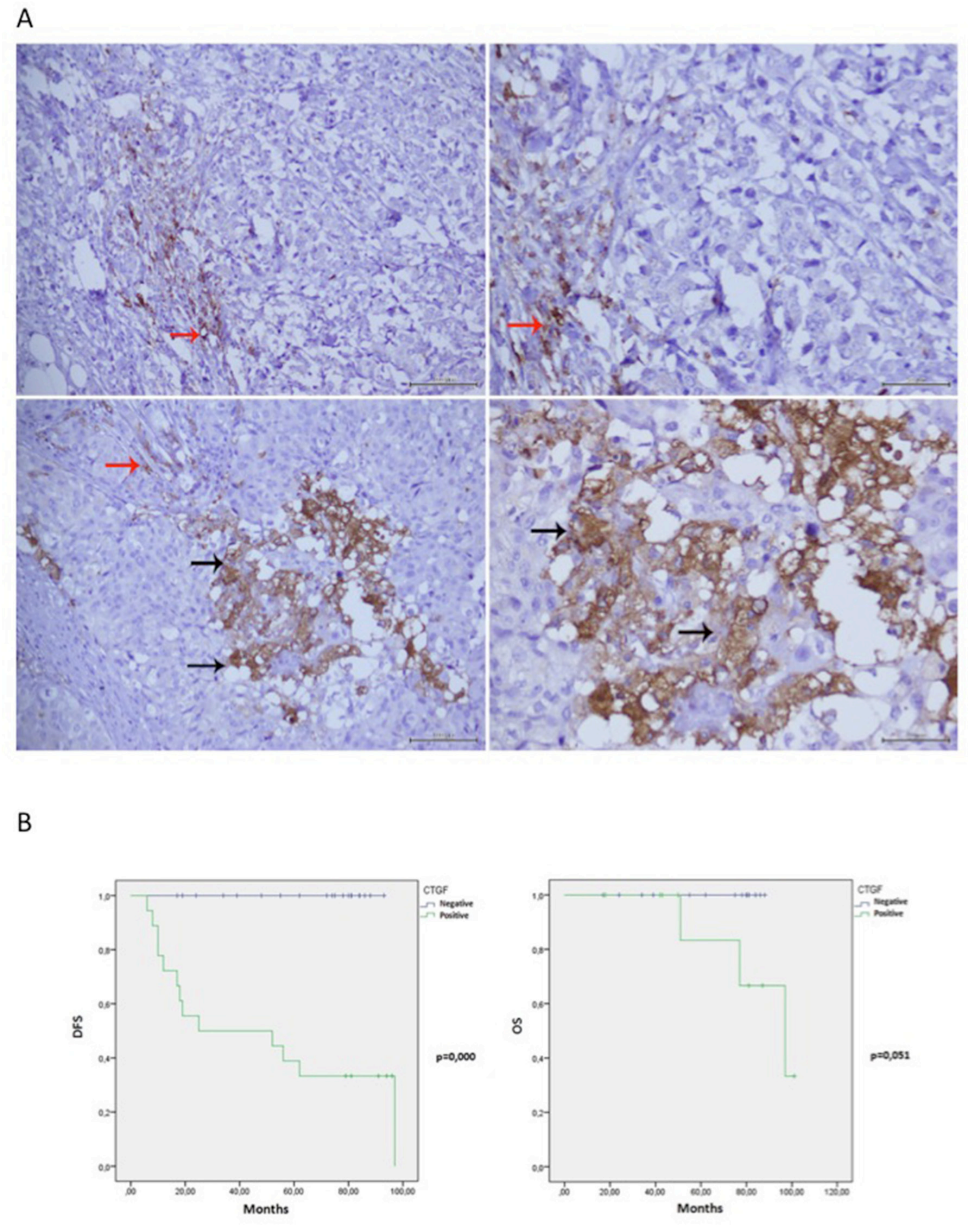
CTGF IHC staining in ER^+^ breast cancer tissues and Kaplan-Meier curves **(A)** Negative staining of CTGF in cancer cells at 20x and 40x magnification (Upper panels, respectively). Positive staining of CTGF in cancer cells (black arrows) at 20x and 40x magnification (Lower panels, respectively). Details of inflammatory cells CTGF staining (red arrows) represented positive internal control. **(B)** Kaplan-Meier curves representing overall survival (OS) and disease-free survival (DFS), have been calculated as described in Experimental Procedures. The follow-up duration was five years. Patients which stained positive for CTGF were reported as Green line; patients which stained negative as blue line.

BC specimens (n=40) obtained from postmenopausal woman were analyzed. The patients’ mean age was 62 years (range from 40 to 79). Tumors larger than 2 cm occurred in 28.2% (11/39) of patients. 42.5% (17/40) had tumor with moderately differentiated cells (grade 2), while 57.5% (23/40) of patients showed a tumor with poorly differentiated cells (grade 3). None of the tumors was of grade 1. Metastatic lymph nodes were found in 42.5% (17/40) of patients at surgery and 35% (14/40) of patients developed distant metastases. The expression of the proliferation factor Ki67 was low (≤20%) in 52.5% (21/40) and high (>20%) in 47.5% (19/40) of specimens. 35.8% (14/39) of patients were diabetic and 87.1% (34/39) were overweight, having a body mass index greater than 25 (Table 2). About 50% (19/40) were positive to CTGF staining and included both ductal and non ductal BC. Statistical analysis showed that CTGF staining in cancer cells (Figure 7A) was not associated with patient age, BMI, fasting glucose levels, diagnosis of diabetes, tumor histotype, size and grading. A slight, although not significant, association with Ki67 proliferation index (p=0.059) and lymph node metastases (p=0.061) was detected. Interestingly, CTGF immune-staining was significantly associated with hormone therapy resistance (p=0.000) and distant metastases (p=0.000) (Table 2). Consistently, Kaplan-Meier curves showed a negative association between CTGF expression and disease free survival (p=0.000) and overall survival (p=0.051) (Figure 7B).

**Table 1:** Primer pairs

*Gene*	*Primer forward*	*T*_*m*_ *F*	*Primer reverse*	*T*_*m*_ *R*	*Product size*
CYR61	TGCGGCTGCTGTAAGGTC	58°C	ACAGAGGAATGCAGCCCAC	60°C	253 bp
EGR3	AAGCTGCCGGTGACCATGA	60°C	GTAGGTCACGGTCTTGTTGC	62°C	234 bp
JUN	TCCAGCAACGGGCACATCA	60°C	GTTGCTGAGGTTTGCGTAGA	60°C	279 bp
EGR2	CCCCTTTGACCAGATGAAC	58°C	TGGATGAGGCTGTGGTTGA	58°C	266 bp
E2F2	CCTCTCCCCTCTACCTCCA	62°C	CAGGTCCCCAAGGTCGTAG	62°C	354 bp
E2F7	CAGGTCCCCAAGGTCGTAG	60°C	GGGACAGTCGGGTTCAGAG	62°C	303 bp
CTGF	GGGAAATGCTGCGAGGAGT	58°C	GATAGGCTTGGAGATTTTGG	58°C	237 bp
IL8	TTCCAAGCTGGCCGTGGCTC	66°C	TGTGTTGGCGCAGTGTGGTCC	68°C	187 bp
HPRT	TGGCGTCGTGATTAGTGATG	60°C	CCCATCTCCTTCATCACATC	60°C	156 bp
PPIA	TACGGGTCCTGGCATCTTGT	62°C	GGTGATCTTCTTGCTGGTCT	60°C	196 bp

List of primer pairs used for quantitative Real-Time PCR.

**Table 2:** Clinical and pathological features of ER^+^ BC samples

*CTGF*				
Age (n=40)	Negative	Positive	p value	R Pearson
≥40≤60	7 (58.3%)	5 (41.7%)	0.629	0.076
>60	14 (50%)	14 (50%)		
Histotype (n=40)				
Ductal	12 (52.2%)	11 (47.8%)	0.962	- 0.008
No Ductal	9 (52.9%)	8 (47.1%)		
Tumor size (n=39)				
≤2 cm	15 (53.6%)	13 (46.4%)	0.956	-0.009
>2≤5	6 (54.5%)	5 (45.5%)		
Limph Node Metastasis (n=40)				
Negative	15 (65.2%)	8 (34.8%)	0.061	0.296
Positive	6 (35.3%)	11 (64.7%)		
Metastases (n=40)				
Negative	20 (76.9%)	6 (33.1%)	0.000^**^	0.666
Positive	1 (7.1%)	13 (92.9%)		
Grade (n=40)				
G2	11 (64.7%)	6 (35.3%)	0.184	0.068
G3	10 (43.5%)	13 (56.5%)		
Ki-67 (n=40)				
≤20%	14 (66.7%)	7 (33.3%)	0.059	0.298
>20%	7 (36.8%)	12 (63.2%)		
BMI (n=39)				
≤25	4 (80%)	1 (20%)	0.169	0.220
>25	16 (47%)	18 (53%)		
Diabetes (n=39)				
No	13 (52%)	12 (48%)	0.905	0.019
Yes	7 (50%)	7 (50%)		
Glycemia (n=39)				
<100	10 (58.8%)	7 (41.2%)	0.408	0.133
≥100	10 (45.5%)	12 (54.5%)		
Status (n=32)				
Alive	16 (66.7%)	8 (33.3%)	0.001^**^	0.577
Dead	0 (0%)	8 (100%)		
Hormone Therapy Resistance (n=40)				
No	21 (77.8%)	6 (22.2%)	0.000^**^	0.729
Yes	0 (0%)	13 (100%)		

Clinical features of the patients with ER^+^ BC which have been enrolled in the study.

## DISCUSSION

T2D and hyperglycemia promote poorer therapeutic outcomes and reduced drug response in BC [7, 14, 25]. Glucose may affect cancer responsiveness to chemotherapy, both directly acting on tumor cells and indirectly affecting surrounding and distant cells. Adipocytes may integrate metabolic derangements (i.e. hyperglycaemia) and drive an indirect influence on several aspects of tumorigenesis, including drug resistance. Tamoxifen has been the mainstay of endocrine therapy in ER^+^ BC. However, approximately 40% of patients with ER^+^ BC do not respond to tamoxifen treatment. Further, most tumors eventually acquire tamoxifen resistance [26]. In this study, both direct and adipose tissue-mediated effect of glucose on ER^+^BC cell responsiveness to tamoxifen have been explored. We demonstrated that BC cells cultured in a glucose concentration resembling hyperglycemia (HG) in humans are less sensitive to tamoxifen than cells grown in a concentration corresponding to normal fasting glucose levels (LG). By using NGS technology to profile the entire cell transcriptome, this study revealed that glucose deregulates cell cycle-related genes. Combining NGS data to gene silencing we provide evidence that the effect of glucose on BC cell sensitivity to tamoxifen is likely mediated by *CTGF*. Specifically, *CTGF* silencing induced a significant increase in tamoxifen sensitivity of BC cells grown in HG, at levels similar to those obtained for cells cultured in LG.

*CTGF* encodes a growth factor, belonging to the CCN protein family. Given its ability to interact with a broad range of different proteins, it enables to transmit cell-specific biological and molecular functions highly dependent on the context. Thus, CTGF has been defined as a “multifunctional matrix-cellular protein” [27]. Among its various functions, it is involved in tumor development and progression and in tumor survival [28]. Although the relationship between CTGF and cancer progression cannot be generalized across different types of cancer [29], CTGF expression is high in steroid-dependent breast tumors [24, 30]. Particularly, CTGF enhances clonogenic ability, cell viability and migration of BC cells [31, 32]. Of note, CTGF confers resistance to doxorubicin- and paclitaxel-induced apoptosis in BC cells [33].

Here, we show, for the first time, that BC cell responsiveness to tamoxifen inversely correlates with *CTGF* expression, providing additional clues to the hypothesis that CTGF is one relevant contributor to drug sensitivity in BC.

Numerous studies highlighted CTGF as possible target in various diseases, including ovarian cancer, pancreatic cancer, osteosarcoma and breast cancer [28, 31, 34, 35]. FG-3019 is a monoclonal antibody against CTGF and is currently under clinical investigation as a therapeutic agent for pancreatic cancer [34, 35]. Interestingly, the treatment with FG-3019 inhibited growth of tumor xenograft and metastases, without exhibiting noticeable side effects, in a model of pancreatic cancer [27].

Of note, a higher expression of CTGF has been also shown in diabetes [30]. CTGF is strongly up-regulated in disorders, frequently developed by diabetic patients, like cardiovascular disease, nephropathy, neuropathy, and retinopathy [36]. In line with this, CTGF has been reported as strongly expressed in glomeruli of diabetic patients and animals with nephropathy [37]. Thus, CTGF is involved in the pathogenesis of chronic inflammatory fibro-proliferative alterations. Consistently, besides the up-regulation of anti-apoptotic (i.e. Survivin) and proliferative genes (Cyclins A2 and B1), CTGF is able to enhance the expression of inflammatory cytokines, including IL8 [26, 38].

We previously demonstrated that glucose enhances the ability of adipocytes to produce factors involved in the control of BC cell proliferation and invasiveness [15, 16]. Therefore, we hypothesized that glucose could exert its effect on BC cell responsiveness to tamoxifen also through an adipocyte-mediated mechanism. We observed that glucose-induced adipocyte-released factors promote a significant over-expression of *CTGF* in BC cells and, in parallel determine a reduction of tamoxifen responsiveness. Adipocytes exposed to high glucose release a higher amount of several cytokines, chemokines and growth factors, including IL8 [15]. In this study, we demonstrated that blocking IL8 with a neutralizing antibody prevents, at least in part, the effect of adipocytes on BC cells in terms of both *CTGF* expression and tamoxifen responsiveness. Silencing *CTGF* in BC cells reduces adipocyte-effect on tamoxifen responsiveness, as well. Thus, *CTGF* regulation is exerted by adipocyte-released IL8, suggesting a reciprocal interplay between adipocytes and cancer cells.

IL8 is a pro-inflammatory cytokine whose expression is regulated by a number of different *stimuli* including inflammatory signals, chemical and environmental stresses and steroid hormones [39]. IL8 *per se* may contribute to cancer growth by its mitogenic and angiogenic action. High serum levels of IL8 in BC patients have been associated with poor prognosis [38]. Targeting IL8 signaling in tumor microenvironment has favorable outcome with regard to halting tumor progression and increasing sensitivity to clinically useful chemotherapy agents in several solid tumors [17, 39]. Reparixin is a small molecular weight inhibitor of IL8 receptors. The drug was in clinical trials for type 1 diabetes, kidney diseases and, nowadays, it is undergoing clinical trials in combination with paclitaxel for metastatic triple negative BC (w).

**Figure 8:**
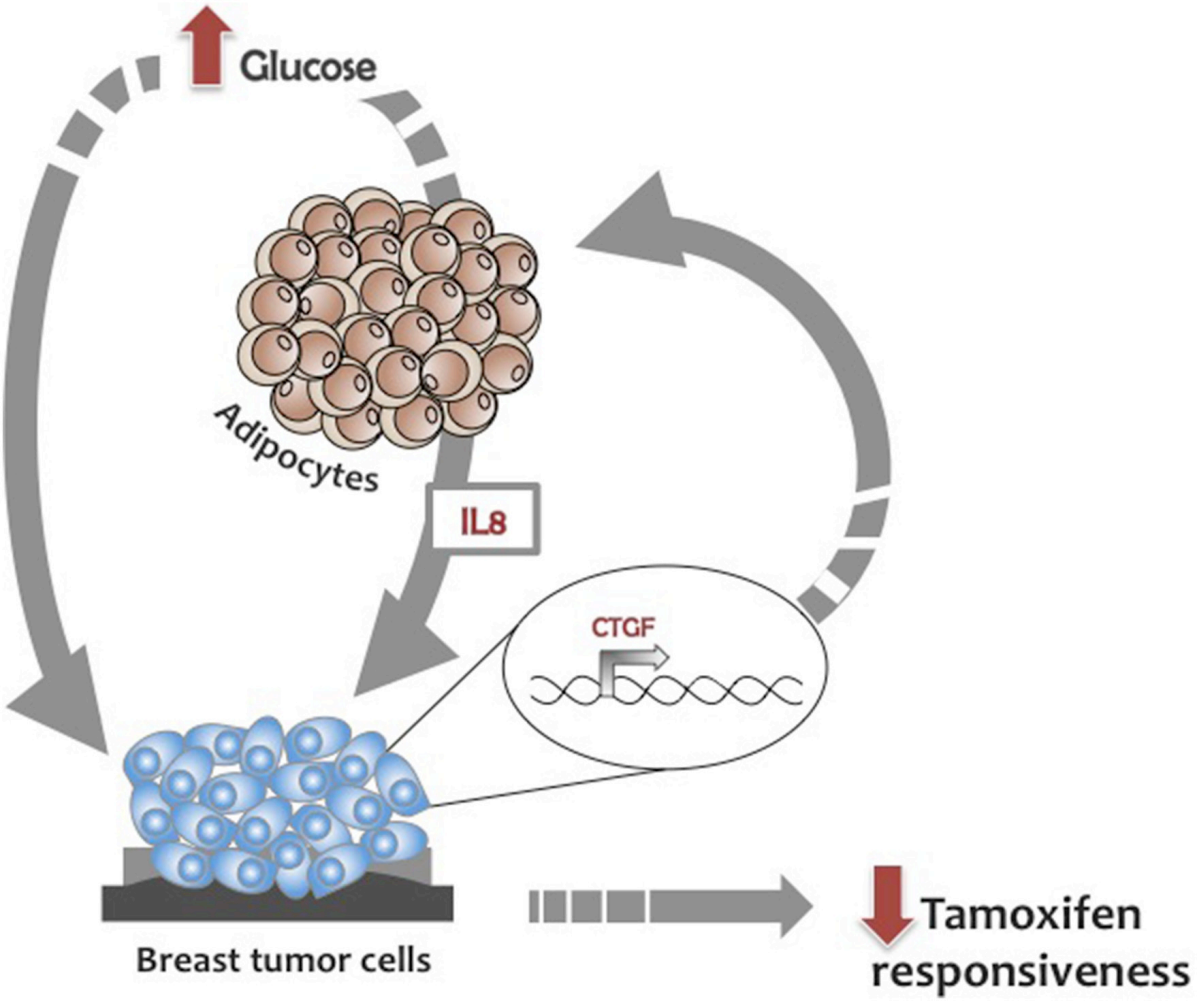
Glucose regulation of BC cell responsiveness to tamoxifen Glucose reduces tamoxifen responsiveness inducing *CTGF* expression in BC cells. Glucose promotes the adipocytes’ release of IL8 that in turn impairs BC cell responsiveness to tamoxifen modulating *CTGF*.

Thus, our hypothesis is that high glucose levels reduce tamoxifen responsiveness up-regulating *CTGF* in BC cells. Of note, glucose levels could affect tamoxifen responsiveness in BC cells also through an adipose-tissue mediated mechanism. Specifically, glucose-induced adipocyte-released IL8 [15] promotes *CTGF* expression in BC cells further impairing their responsiveness to tamoxifen treatment (Figure 8).

Interestingly, we now describe that CTGF correlates with hormone therapy resistance in women with ER^+^ BC. Moreover, patients positive to CTGF display higher levels of metastatic dissemination and decreased survival rate. No direct correlation was found between CTGF staining in BC and the presence of diabetes or hyperglycemia. However, high levels of CTGF have been reported in diabetic complications [36, 37]. Of note, 6 out of 7 diabetic patients positive to CTGF staining were obese. Conversely, none of normal weight and euglycemic individuals were positive to CTGF staining. Thus, we could speculate that other *stimuli* in addition to glucose can also enhance CTGF expression in BC cells, thereby contributing to tamoxifen resistance and tumor dissemination.

In conclusion, CTGF may be considered as predictive marker for chemo-sensitivity and potential therapeutic target to overcome tamoxifen resistance. Moreover, targeting IL8 in tumor microenvironment could not only reduce the inflammatory state but also indirectly modulate CTGF, thus improving the effectiveness of tamoxifen treatment in BC.

## MATERIALS AND METHODS

### Materials

Media, sera and antibiotics for cell culture were from Lonza (Basel, Switzerland). Human recombinant CTGF and IL8 proteins were from PeproTech (Rocky Hill, NJ, USA). Policlonal antibodies against CTGF and Actin were purchased from Santa Cruz Biotechnology (Santa Cruz Biotechnology, Santa Cruz, CA, USA). Secondary anti-goat antibody AlexaFluor 488 was from Abcam (Cambridge, UK). Monoclonal antibodies against human IL8 and CCL5 were from Abcam (Cambridge, UK). CCR5 peptide (CCR5-pep) was a gift from Prof. P. Campiglia (Department of Pharmacy, University of Salerno, Italy). Synthesis, purification and characterization of CCR5-pep (Sequence: AFDWTFVPSLIL-NH2) were described by D’Esposito [16]. Monoclonal/Polyclonal antibodies against human ERα, PR (Clone ID5), c-Erb B2 and Ki67 Ag (Clone MIB-1) were from DAKO (Ely, UK). All the other chemicals were from Sigma-Aldrich (St Louis, MO, USA).

### Cell cultures

MCF7 human breast cancer cells (ERɑ^+^, PR^+^, HER2^-^) were cultured in Dulbecco’s modified Eagle’s medium (DMEM) supplemented with 10% fetal bovine serum (FBS), 2 mM glutamine, 100 units/ml penicillin and 100 units/ml streptomycin. 48 hours before the induction of the experiments, the cells were shifted to phenol red free DMEM supplemented with 10% Charcoal Stripped (C/S) FBS, 2 mM glutamine, 100 units/ml penicillin and 100 units/ml streptomycin. Cultures were maintained in a humidified atmosphere of 95% air and 5% CO_2_ at 37°C.

Human adipose tissue samples were obtained from mammary adipose biopsies of healthy women (n=12; age 25-63 years; BMI 24.2 – 29.0) undergoing surgical mammary reduction. All women were otherwise healthy and free of metabolic or endocrine diseases. Informed consent was obtained from every subject before the surgical procedure. Such procedure was approved by the ethical committee of the University of Naples “Federico II”. Adipose derived Mesenchymal Stem Cells (Ad-MSCs) were isolated from the Stromal Vascular Fraction and differentiated in mature adipocytes as previously described [15, 40].

### Sulforhodamine B assay

MCF7 cells were fixed with 50% trichloroacetic acid for at least 2 hours at 4°C, washed with distilled and de-ionized water, air-dryed and stained 30 minutes with 0.4% sulforhodamine B in 1% acetic acid. Unbound dye was removed and 10mM Tris solution (pH 7.5) was added to dissolve the protein-bound dye. Cell survival was assessed by optical density determination at 510 nm using a microplate reader.

### RNA isolation and analysis

Total RNA was isolated from cells using TRIzol solution (Life Technologies, Carlsbad, CA, USA) according to the manufacturer's instructions. All RNA samples were quantified by measuring the absorbance at 260nm and 280nm (NanoDrop spectrophotometer, Life Technologies, Carlsbad, CA, USA). The integrity of RNA samples was further analyzed by using the digital electrophoresis system Experion with the “RNA StdSens Kit” (Biorad, Hercules, CA, USA), following the manufacturer's instructions. The run and result analysis were performed by the Experion software. RNA samples with a RNA Quality Indicator (RQI) value ≥ 9 were considered good for the further analysis.

### RNA-Sequencing

#### cDNA libraries preparation

Paired-end cDNA libraries were prepared for sequencing on the Illumina Hi-Seq 2000 platform using “TruSeq RNA Sample Preparation kit” (Illumina) according to manufacturer's protocol [41]. The yield and the size distribution of purified cDNA libraries were assessed using “Qubit dsDNA HS (High Sensitivity) Assay Kit” on Qubit Fluorometer (Life Technologies, Carlsbad, CA, USA) and “Experion DNA 1K Analysis Kit” (Biorad, Hercules, CA, USA) on the Experion system (Biorad, Hercules, CA, USA), respectively. Peak median values were determined at approximately 200-260bp. Sample concentration and peak median values were used to calculate molar concentration of cDNA libraries prior to sequencing on the Illumina HiSeq 2000, available at Tigem Institute (Pozzuoli, Italy).

#### Data analysis

Reads' quality was assessed using “FastQC” and TopHat software (version 2.0.10) [42]. was used to map the reads against the RefSeq human transcripts and reference human genome (UCSC hg19 release) with default parameters. RefSeq annotation was downloaded from UCSC Genome Browser (http://genome.ucsc.edu) and only the protein-coding gene annotation coordinates were used. Uniquely mapping reads (about 90% of sequenced reads) were used for further analyses. SAMTools and BEDTools were used to convert file formats (.sam/.bam/.bed). Coverage files in bedgraph format were produced using the GenomeCoverage option of BEDTools. Filtering of low-coverage genes (Proportion test method), normalization (upper quartile method), quantification of gene expression (SummerizeOverlaps option), and the analysis of differential expression (NoiSeq method) were carried out using “RNASeqGUI” [43]. A posterior probability threshold of 0.8 was used to select differentially expressed genes (DEGs). Pathway analysis was performed using PANTHER (Protein ANalysis THrough Evolutionary Relationships; http://www.pantherdb.org).

### RT-PCR

RNA samples (1μg) were reverse transcribed using SuperScript II Reverse Transcriptase with oligo dT primers (Invitrogen, Carlsbad, CA, USA) according to the manufacturer's instructions. To check the amplifiable template RNA/cDNA, RT-PCR amplification of housekeeping genes (Hypoxanthine Guanine Phosphoribosyl Transferase - *HPRT*; Peptidylprolyl Isomerase A – *PPIA*) was performed in all samples. Each amplification reaction was set-up using AmpliTaq Gold (Life Technologies, Carlsbad, CA, USA) and specific primer pairs, designed using Oligo 4.0 (Table 1). PCR products were analyzed by electrophoresis on agarose gel and Sanger sequencing.

### Quantitative real time RT-PCR

Quantitative Real-Time RT-PCRs (qReal-Time RT-PCR) were performed by iTaq Universal SYBR Green Supermix (Biorad, Hercules, CA, USA), according to the manufacturer’s instructions for the CFX Connect Real Time system (Biorad, Hercules, CA, USA). Relative quantification of gene expression was measured by using 2^−ΔΔCt^ method. Expression levels were normalized for the reference sample using *HPRT* or *PPIA* as housekeeping gene.

### Cell transfection

MCF7 cells were transfected with Dicer-substrate RNAs (DsiRNAs, IDT Coralville, Iowa, USA) by using Lipofectamine 3000 (Life Technologies, Carlsbad, CA, USA), in DMEM without antibiotics and serum, according to manufacturer's instructions. After 6 hours, the cells were feed with phenol red free medium supplemented with 10% C/S FBS and stimulated for further 24 hours.

### Cytofluorimetric analysis

MCF7 cells were incubated (30 minutes at 4°C) with each antibody following treatment for 12 hours with GolgiStop solution (BD Biosciences, Mississauga, ON, Canada) and cell permeabilization with Inside Stain Kit (Miltenyi, Bergisch Gladbach, Germany). Cells were analyzed with a FACS Calibur cytofluorimeter using CellQuest software (BD Biosciences). 10^4^ events for each sample were acquired in all analyses [44].

### Conditioned media system

Mature adipocytes were washed two times with sterile phosphate-buffered saline (PBS) and incubated with serum-free DMEM supplemented with 0.25% albumin bovine serum (BSA). After 8 hours, adipocyte-conditioned media (hAdipo-CM) were collected, centrifuged to remove cellular debris and placed onto recipient cells.

### Co-culture assay

Adipocyte differentiation of Ad-MSCs was carried out in the bottom chamber of a transwell culture system (0.4μm pore size, Costar, Cambridge, MA, USA). At the end of the adipogenic process, MCF7 cells were seeded in the upper chamber. After 24 hours, cell viability was determined by crystal violet staining. Briefly, MCF7 cells were fixed with 11% glutaraldehyde for 15 minutes at RT. Then, cells were washed with PBS and stained with 0.1% crystal violet-20% methanol 20 minutes at RT. After further washes and complete drying, crystal violet was solubilized in 10% acetic acid. The concentration of the solubilized crystal violet was evaluated by optical density determination at 540 nm using a microplate reader.

### Immunohistochemistry analysis

#### Patients and specimens

From 2007 to 2010, 40 patients who underwent a mastectomy or quadrantectomy at the National Cancer Institute “G. Pascale Foundation” of Naples (Italy) were enrolled. All specimens were characterized for routinely diagnostic immune-phenotypic parameters and showed positive staining for the ER^+^. Medical records for all ER^+^ samples were reviewed for clinical information; histologic parameters were determined from the H&E slides. The following clinical and pathological parameters were evaluated for each tumor included in the study: patient age at initial diagnosis, tumor size, histologic subtype, nuclear grade, number of metastatic lymph nodes, size of tumor, tumor recurrence and distant metastasis.

#### Immunohistochemical staining and evaluation

Immunohistochemistry was performed on slides from formalin-fixed, paraffin-embedded tissues, as previously described [16]. The slides were incubated with primary antibody to human CTGF (Santa Cruz Biotechnology) (dilution 1:30) over night and with human ERα, PR, c-Erb B2, Ki67 antibodies for 30 minutes.

Antigen expression of CTGF was independently evaluated by three pathologists (MDB, GB and RF) by using a light microscopy. Observers was unaware of the clinical outcome. For each sample, at least five HPF (High Power Fields) and >500 cells were analyzed. Since standardized criteria for CTGF staining evaluation were not defined, using a semiquantitative scoring system microscopically and referring to each antigen scoring method in other studies, we evaluated the intensity, extent and subcellular distribution of CTGF marker, evaluating cytoplasmic CTGF protein levels in cancer cells and considering only the positive or negative staining. CTGF detection in inflammatory cells was considered as internal positive control. The proliferative index Ki67 was defined as the percentage of immunoreactive tumour cells out of the total number of cells. The percentage of positive cells per case was scored according to 2 different groups: group 1: <20% (low proliferative activity); group 2: ≥20% (high proliferative activity). Other routinely diagnostic markers (ERα, PR and c-Erb B2) were evaluated as previously described [45].

### Statistical analysis

The Pearson χ^2^ test was used to determine the association between CTGF and clinical pathological features included in the study. The level of significance was defined as p<0.05. Overall survival (OS) and disease-free survival (DFS) curves were calculated using the Kaplan-Meier method with significance evaluated using the Mantel-Cox log-rank test. OS was defined as the time from diagnosis (first biopsy) to death by any cause or until the most recent follow-up. DFS was measured as the time from diagnosis to the occurrence of progression, relapse after complete remission, or death from any cause. DFS had a value of zero for patients who did not achieve complete remission. The follow-up duration was five years. All the statistical analyses were carried out using the Statistical Package for Social Science v.20 software (SPSS Inc., Chicago, IL, USA).

## SUPPLEMENTARY MATERIALS FIGURES



## References

[1] Maccioò A, Madeddu C, Mantovani G (2009). Adipose tissue as target organ in the treatment of hormone-dependent breast cancer: new therapeutic perspectives. Obes Rev.

[2] Tsilidis KK, Kasimis JC, Lopez DS, Ntzani EE, Ioannidis JP (2015). Type 2 diabetes and cancer: umbrella review of meta-analyses of observational studies. BMJ.

[3] Khandekar MJ, Cohen P, Spiegelman BM (2011). Molecular mechanisms of cancer development in obesity. Nat Rev Cancer.

[4] Zeng L, Biernacka KM, Holly JM, Jarrett C, Morrison AA, Morgan A, Winters ZE, Foulstone EJ, Shield JP, Perks CM (2010). Hyperglycaemia confers resistance to chemotherapy on breast cancer cells: the role of fatty acid synthase. Endocr Relat Cancer.

[5] Barone BB, Yeh HC, Snyder CF, Peairs KS, Stein KB, Derr RL, Wolff AC, Brancati FL (2010). Postoperative mortality in cancer patients with preexisting diabetes: systematic review and meta-analysis. Diabetes Care.

[6] Renehan AG, Yeh HC, Johnson JA, Wild SH, Gale EA, Møller H, Diabetes and Cancer Research Consortium (2012). Diabetes and cancer (2): evaluating the impact of diabetes on mortality in patients with cancer. Diabetologia.

[7] Zeng L, Zielinska HA, Arshad A, Shield JP, Bahl A, Holly JM, Perks CM (2016). Hyperglycaemia-induced chemoresistance in breast cancer cells: role of the estrogen receptor. Endocr Relat Cancer.

[8] Hanahan D, Weinberg RA (2011). Hallmarks of cancer: the next generation. Cell.

[9] O'Mahony F, Razandi M, Pedram A, Harvey BJ, Levin ER (2012). Estrogen modulates metabolic pathway adaptation to available glucose in breast cancer cells. Mol Endocrinol.

[10] Gallagher EJ, LeRoith D (2015). Obesity and diabetes: the increased risk of cancer and cancer-related mortality. Physiol Rev.

[11] Duan W, Shen X, Lei J, Xu Q, Yu Y, Li R, Wu E, Ma Q (2014). Hyperglycemia, a neglected factor during cancer progression. Biomed Res Int.

[12] Quail DF, Joyce JA (2013). Microenvironmental regulation of tumor progression and metastasis. Nat Med.

[13] Balkwill FR, Capasso M, Hagemann T (2012). The tumor microenvironment at a glance. J Cell Sci.

[14] Calle EE, Kaaks R (2004). Overweight, obesity and cancer: epidemiological evidence and proposed mechanisms. Nat Rev Cancer.

[15] D'Esposito V, Passaretti F, Hammarstedt A, Liguoro D, Terracciano D, Molea G, Canta L, Miele C, Smith U, Beguinot F, Formisano P (2012). Adipocyte-released insulin-like growth factor-1 is regulated by glucose and fatty acids and controls breast cancer cell growth *in vitro*. Diabetologia.

[16] D'Esposito V, Liguoro D, Ambrosio MR, Collina F, Cantile M, Spinelli R, Raciti GA, Miele C, Valentino R, Campiglia P, De Laurentiis M, Di Bonito M, Botti G (2016). Adipose microenvironment promotes triple negative breast cancer cell invasiveness and dissemination by producing CCL5. Oncotarget.

[17] Duong MN, Cleret A, Matera EL, Chettab K, Mathé D, Valsesia-Wittmann S, Clémenceau B, Dumontet C (2015). Adipose cells promote resistance of breast cancer cells to trastuzumab-mediated antibody-dependent cellular cytotoxicity. Breast Cancer Res.

[18] Yi EH, Lee CS, Lee JK, Lee YJ, Shin MK, Cho CH, Kang KW, Lee JW, Han W, Noh DY, Kim YN, Cho IH, Ye SK (2013). STAT3-RANTES autocrine signaling is essential for tamoxifen resistance in human breast cancer cells. Mol Cancer Res.

[19] Knowlden JM, Hutcheson IR, Jones HE, Madden T, Gee JM, Harper ME, Barrow D, Wakeling AE, Nicholson RI (2003). Elevated levels of epidermal growth factor receptor/c-erbB2 heterodimers mediate an autocrine growth regulatory pathway in tamoxifen-resistant MCF-7 cells. Endocrinology.

[20] Gutierrez MC, Detre S, Johnston S, Mohsin SK, Shou J, Allred DC, Schiff R, Osborne CK, Dowsett M (2005). Molecular changes in tamoxifen-resistant breast cancer: relationship between estrogen receptor, HER-2, and p38 mitogen-activated protein kinase. J Clin Oncol.

[21] Peifer C, Alessi DR (2009). New anti-cancer role for PDK1 inhibitors: preventing resistance to tamoxifen. Biochem J.

[22] Mojarrad M, Momeny M, Mansuri F, Abdolazimi Y, Tabrizi MH, Ghaffari SH, Tavangar SM, Modarressi MH (2010). Autocrine human growth hormone expression leads to resistance of MCF-7 cells to tamoxifen. Med Oncol.

[23] Huber-Keener KJ, Liu X, Wang Z, Wang Y, Freeman W, Wu S, Planas-Silva MD, Ren X, Cheng Y, Zhang Y, Vrana K, Liu CG, Yang JM, Wu R (2012). Differential gene expression in tamoxifen-resistant breast cancer cells revealed by a new analytical model of RNA-Seq data. PLoS One.

[24] Xie D, Nakachi K, Wang H, Elashoff R, Koeffler HP (2001). Elevated levels of connective tissue growth factor, WISP-1, and CYR61 in primary breast cancers associated with more advanced features. Cancer Res.

[25] Park J, Morley TS, Kim M, Clegg DJ, Scherer PE (2014). Obesity and cancer- mechanisms underlying tumour progression and recurrence. Nat Rev Endocrinol.

[26] Moriai R, Tsuji N, Moriai M, Kobayashi D, Watanabe N (2009). Survivin plays as a resistant factor against tamoxifen-induced apoptosis in human breast cancer cells. Breast Cancer Res Treat.

[27] Seher A, Nickel J, Mueller TD, Kneitz S, Gebhardt S, ter Vehn TM, Schlunck G, Sebald W (2011). Gene expression profiling of connective tissue growth factor (CTGF) stimulated primary human tenon fibroblasts reveals an inflammatory and wound healing response *in vitro*. Mol Vis.

[28] Shi-Wen X, Leask A, Abraham D (2008). Regulation and function of connective tissue growth factor/CCN2 in tissue repair, scarring and fibrosis. Cytokine Growth Factor Rev.

[29] Tsai HC, Huang CY, Su HL, Tang CH (2014). CTGF increases drug resistance to paclitaxel by upregulating survivin expression in human osteosarcoma cells. Biochim Biophys Acta.

[30] Brigstock DR (2003). The CCN family: a new stimulus package. J Endocrinol.

[31] Chen PS, Wang MY, Wu SN, Su JL, Hong CC, Chuang SE, Chen MW, Hua KT, Wu YL, Cha ST, Babu MS, Chen CN, Lee PH (2007). CTGF enhances the motility of breast cancer cells via an integrin-alphavbeta3-ERK1/2-dependent S100A4-upregulated pathway. J Cell Sci.

[32] Chien W, O'Kelly J, Lu D, Leiter A, Sohn J, Yin D, Karlan B, Vadgama J, Lyons KM, Koeffler HP (2011). Expression of connective tissue growth factor (CTGF/CCN2) in breast cancer cells is associated with increased migration and angiogenesis. Int J Oncol.

[33] Wang MY, Chen PS, Prakash E, Hsu HC, Huang HY, Lin MT, Chang KJ, Kuo ML (2009). Connective tissue growth factor confers drug resistance in breast cancer through concomitant up-regulation of Bcl-xL and cIAP1. Cancer Res.

[34] Moran-Jones K, Gloss BS, Murali R, Chang DK, Colvin EK, Jones MD, Yuen S, Howell VM, Brown LM, Wong CW, Spong SM, Scarlett CJ, Hacker NF (2015). Connective tissue growth factor as a novel therapeutic target in high grade serous ovarian cancer. Oncotarget.

[35] Neesse A, Frese KK, Bapiro TE, Nakagawa T, Sternlicht MD, Seeley TW, Pilarsky C, Jodrell DI, Spong SM, Tuveson DA (2013). CTGF antagonism with mAb FG-3019 enhances chemotherapy response without increasing drug delivery in murine ductal pancreas cancer. Proc Natl Acad Sci U S A.

[36] Lin CH, Wang YH, Chen YW, Lin YL, Chen BC, Chen MC (2016). Transcriptional and posttranscriptional regulation of CXCL8/IL-8 gene expression induced by connective tissue growth factor. Immunol Res.

[37] Roestenberg P, van Nieuwenhoven FA, Wieten L, Boer P, Diekman T, Tiller AM, Wiersinga WM, Oliver N, Usinger W, Weitz S, Schlingemann RO, Goldschmeding R (2004). Connective tissue growth factor is increased in plasma of type 1 diabetic patients with nephropathy. Diabetes Care.

[38] Bendrik C, Dabrosin C (2009). Estradiol increases IL-8 secretion of normal human breast tissue and breast cancer *in vivo*. J Immunol.

[39] Waugh DJ, Wilson C (2008). The interleukin-8 pathway in cancer. Clin Cancer Res.

[40] Ambrosio MR, Aprile M, D'Esposito V, Beguinot F, Formisano P, Costa V, Ciccodicola A (2014). PPARG in human adipogenesis: differential contribution of canonical transcripts and dominant negative isoforms. PPAR Res.

[41] Costa V, Esposito R, Ziviello C, Sepe R, Bim LV, Cacciola NA, Decaussin-Petrucci M, Pallante P, Fusco A, Ciccodicola A (2015). New somatic mutations and WNK1-B4GALNT3 gene fusion in papillary thyroid carcinoma. Oncotarget.

[42] Kim D, Pertea G, Trapnell C, Pimentel H, Kelley R, Salzberg SL (2013). TopHat2: accurate alignment of transcriptomes in the presence of insertions, deletions and gene fusions. Genome Biol.

[43] Russo F, Angelini C (2014). RNASeqGUI: a GUI for analysing RNA-Seq data. Bioinformatics.

[44] Prevete N, Liotti F, Illiano A, Amoresano A, Pucci P, de Paulis A, Melillo RM (2017). Formyl peptide receptor 1 suppresses gastric cancer angiogenesis and growth by exploiting inflammation resolution pathways. Oncoimmunology.

[45] Collina F, Di Bonito M, Li Bergolis V, De Laurentiis M, Vitagliano C, Cerrone M, Nuzzo F, Cantile M, Botti G (2015). Prognostic value of cancer stem cells markers in triple-negative breast cancer. Biomed Res Int.

