# Supplementary material for: Glucose impairs tamoxifen responsiveness modulating connective tissue growth factor in breast cancer cells
Source: Oncotarget. 2017 Nov 20;8(65):109000–17. doi: 10.18632/oncotarget.22552 (PMC5752499; doi:10.18632/oncotarget.22552)
Supplement: Supplementary file 1 [file oncotarget-08-109000-s001.pdf]

Glucose impairs tamoxifen responsiveness modulating connective tissue growth factor in breast cancer cells

SUPPLEMENTARY MATERIALS

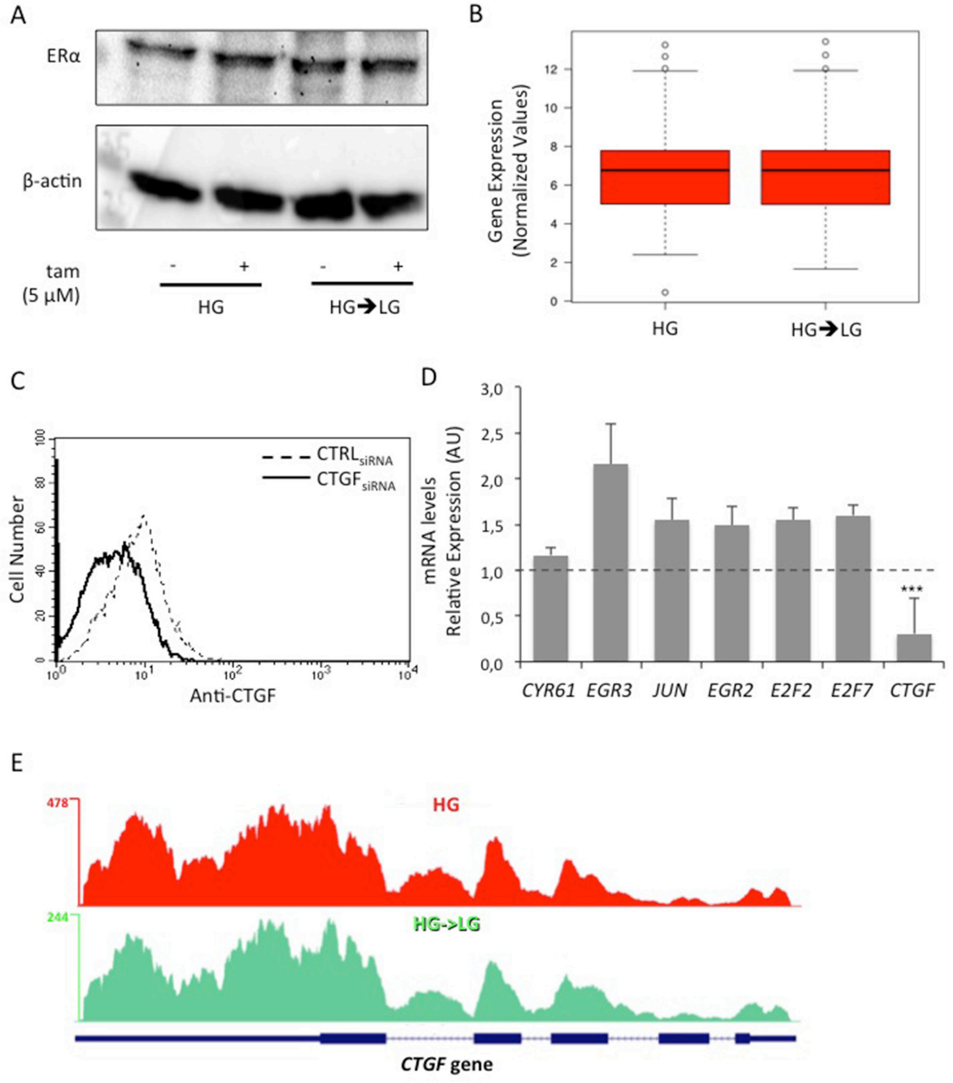

Supplementary Figure 1: Glucose effect on estrogen receptor-α and on whole transcriptome in MCF7 cells. Related to Figures 1, 2 and 3. **(A)** HG and HG→LG cells were treated with E2 (100nM) and tam (5μM) for four days. As positive control, the cells were treated with E2 alone. Cell lysates (50μg protein/sample) were blotted with ERα antibodies. To ensure the equal protein transfer, membranes were blotted with β-actin antibodies. The filters were revealed by ECL and autoradiography. The autoradiographs shown are representative of four independent experiments. **(B)** Boxplot showing the log-transformed distribution of filtered expression data for each condition. **(C)** CTGF protein expression in MCF7 cells transfected with siRNA targeting CTGF ($CTGF_{siRNA}$ - solid line) or control siRNA ($CTRL_{siRNA}$ - dotted line), as evaluated by cytofluorimetric analysis. One representative experiment is shown. **(D)** mRNA levels of "Cell cycle" DEGs in $CTGF_{siRNA}$ cells were determined by qRealTime-PCR. Data were normalized on HPRT gene as internal standard. Bars represent the mean ± SD of three independent triplicate experiments and show the mRNA levels of "Cell cycle" DEGs in $CTGF_{siRNA}$ cells relative to those in $CTRL_{siRNA}$ cells (dotted line). * denote statistically significant values ($^{***}p<0.001$). **(E)** Schematic representation of CTGF expression in HG and HG→LG samples. Colored peaks (red for HG and green for HG→LG) indicate RNA-Seq reads' coverage across CTGF gene in both the conditions. On the left, the maximum coverage per samples is reported. In the lower part of the panel, a graphical representation of CTGF gene is illustrated. Blue boxes indicate the exons, thin lines the introns and arrows indicate the direction of CTGF transcription (3'-5', i.e. strand minus).

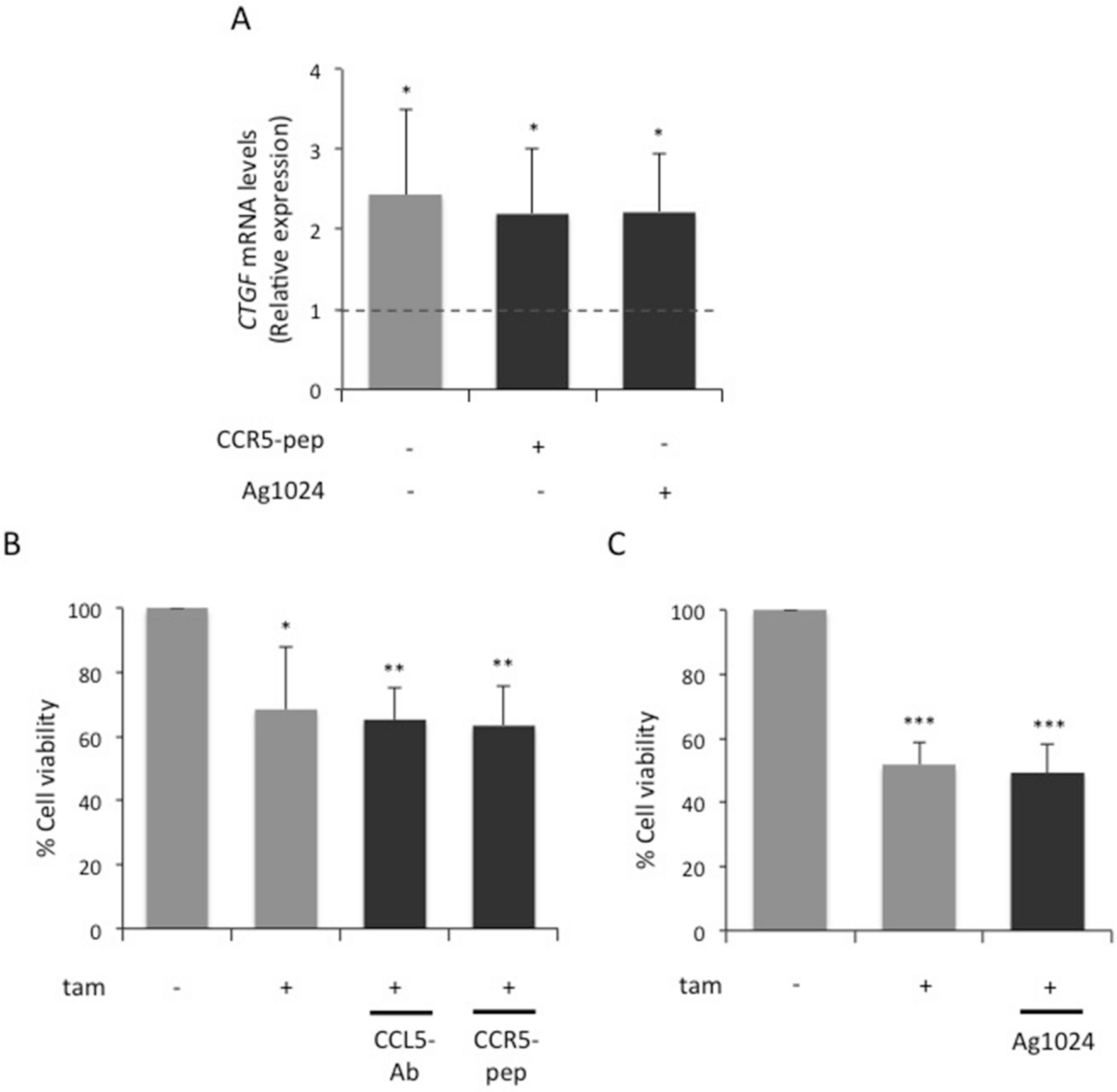

Supplementary Figure 2: Effect of adipocyte-released IGF-1 and CCL5 on MCF7 cell responsiveness to tamoxifen. Related to Figure 5. **(A)** MCF7 cells were incubated with HG hAdipo-CM in presence or absence of a CCL5 receptor (CCR5) antagonist peptide (5µg/ml; CCR5-pep) or a IGF1 receptor tyrosine kinase inhibitor (10µM; Ag1024). As control, the cells were incubated with serum free HG medium. After four days, CTGF mRNA levels were determined by qRealTime-PCR. Data were normalized on HPRT gene as internal standard. Bars represent the mean ± SD of three independent triplicate experiments and show the mRNA levels of CTGF relative to control (dotted line). * denote statistically significant values ($^{*}p<0.05$). **(B)** MCF7 cells were treated with E2 (100nM) and tam (5µM) in presence of HG hAdipo-CM with and without CCL5 Antibody (6µg/ml; CCL5-Ab) or CCR5-pep (5µg/ml; CCR5-pep). **(C)** MCF7 cells were treated with E2 (100nM) and tam (5µM) in presence of HG hAdipo-CM with and without Ag1024 (10µM). For both panels (B) and (C) in the figure, as positive control, the cells were treated with E2 alone. After four days, cell viability was determined by sulforhodamine B assay. The results were reported as percentage of viable cells compared with positive control, considered as 100% viable cells. Data represent the mean ± SD of three independent triplicate experiments. * denote statistically significant values compared with positive control ($^{*}p<0.05$; $^{**}p<0.01$; $^{***}p<0.001$).

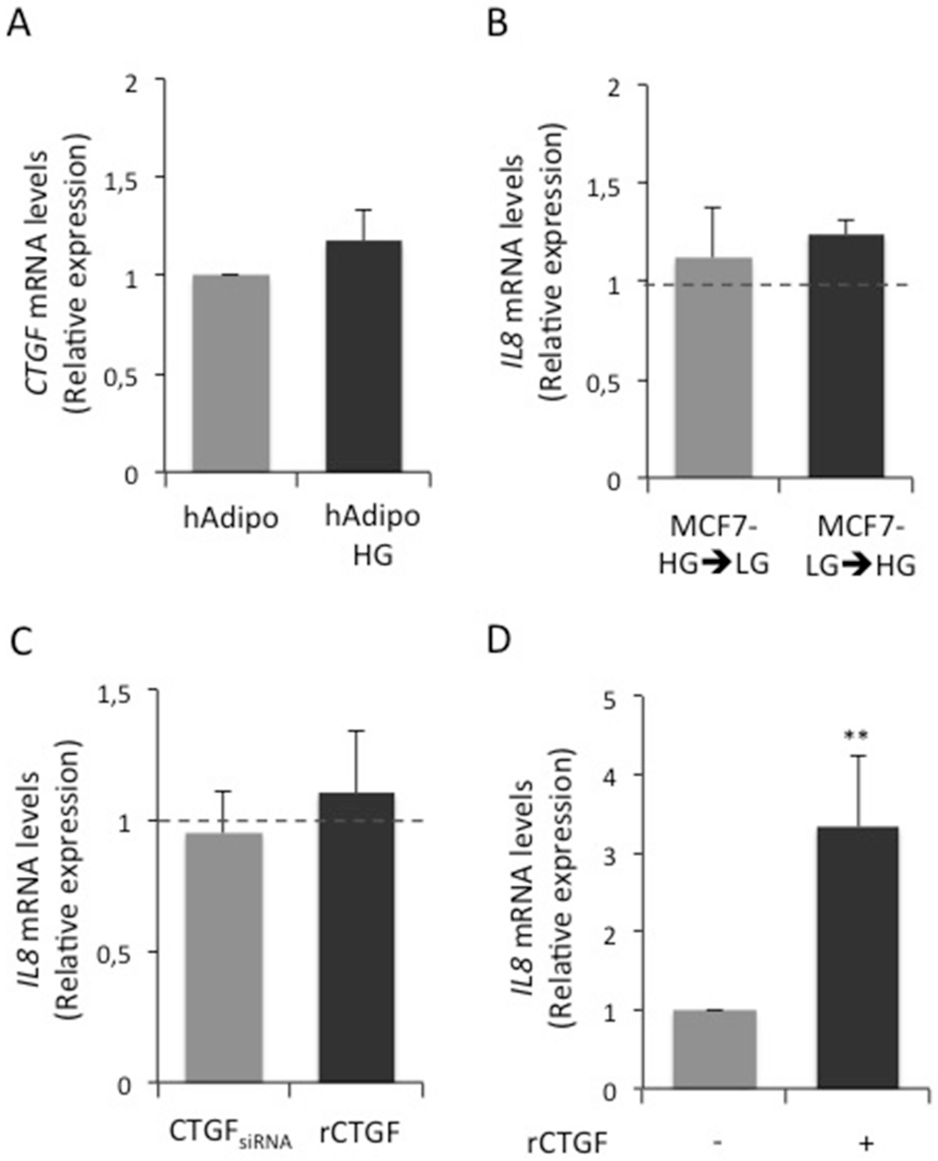

Supplementary Figure 3: Evaluation of the reciprocal effect of IL8 and CTGF in MCF7 and adipocytes. Related to Figure 5. **(A)** mRNA levels of CTGF in mature adipocytes pre-incubated – or not - in high glucose (25mM; HG) medium were measured by qRealTime-PCR. Data were normalized on Peptidylprolyl Isomerase A (PPIA) gene as internal standard. Bars represent the mean ± SD of three independent experiments and show the mRNA levels of CTGF in adipocytes incubated with in HG compared to those in regular medium. **(B)** mRNA levels of IL8 in HG→LG and LG→HG MCF7 cells were determined by qRealTime-PCR. Data were normalized on HPRT gene as internal standard. Bars represent the mean ± SD of three independent triplicate experiments and show the mRNA levels of IL8 in HG→LG and LG→HG cells relative to those in HG and LG cells, respectively. **(C)** mRNA levels of IL8 in MCF7 cell upon CTGF knockdown ($CTGF_{siRNA}$) or rCTGF treatment were determined by qRealTime-PCR. Data were normalized on HPRT gene as internal standard. Bars represent the mean ± SD of three independent triplicate experiments and show the mRNA levels of CTGF in $CTGF_{siRNA}$ cells, relative to those in $CTRL_{siRNA}$ cells, and in cells treated with rCTGF, relative to those in untreated cells. **(D)** Mature adipocytes were incubated with and without human recombinant CTGF protein (1µg/mL; rCTGF) in serum free high glucose medium (25mM, HG). After 24 hours, mRNA expression levels of IL8 were determined by qRealTime-PCR. Data were normalized on PPIA gene as internal standard. Bars represent the mean ± SD of three independent triplicate experiments and show the mRNA levels of IL8 in adipocytes treated with rCTGF relative to those in untreated cells. * denote statistically significant values (**p<0.01).
